# Gender Asymmetries: An Exploratory Study of Women’s Experiences in Portuguese Football Organizations

**DOI:** 10.3390/ejihpe14050081

**Published:** 2024-05-04

**Authors:** Maria Helena Santos, António Manuel Marques, Joana Salvador

**Affiliations:** 1 Instituto Universitário de Lisboa (Iscte-IUL), CIS-Iscte, 1749-016 Lisboa, Portugal; 2 Escola Superior de Saúde do Instituto Politécnico de Setúbal, 2910-761 Setúbal, Portugal; antonio.marques@ess.ips.pt; 3 Instituto Universitário de Lisboa (Iscte-IUL), 1749-016 Lisboa, Portugal; joana_salvador@iscte-iul.pt

**Keywords:** football, tokenism, gender relations, Portuguese women

## Abstract

The main objective of this exploratory study is to analyze the negative effects associated with the phenomenon of tokenism for Portuguese women in non-playing football roles, as well as their strategies for integration into a context where they are a minority and the social functions are dominated by men. To this end, we carried out semi-structured individual interviews with eight men and eight women, undertaking functions in the fields of coaching, psychology, medical team, planning, refereeing, and management in football organizations, and with their contents then subject to thematic analysis. Our analysis confirms how the football context strongly demarcates gender differences with women being more visible and leads to the stereotypical roles being associated with traditional femininity. The findings also observe how, in order to integrate, women align with the norms imposed by the dominant group (men) and accept being circumscribed to the spaces that hegemonic gender ideologies and practices attribute to them.

## 1. Introduction

Despite the transformations and improvements related to the blurring of inequalities and segregation of women in the world of work and professions since the second half of the 20th century [1], there is ample evidence that there are professional contexts that resist these changes and remain under the dominance and predominance of men, and even in those fields experiencing a relative increase in the number of women [2,3]. Contexts characterized by male dominance and the dominance of hegemonic forms of masculinity and in which women constitute a minority have attracted the interest of researchers in keeping with the opportunity to observe and understand the processes of their integration and adaptation [4].

Therefore, the interest in exploring the experiences of men and women in organizational contexts where each represents a minority situation is justified within the scope of deepening the knowledge about the effects of gender and the eventual differentiated consequences of tokenism.

This exploratory study focuses on the context of Portuguese football (staff/non-players), starting out with the expectation that, due to their under-representation in organizations of that sport, in which there will be a culture guided by hegemonic masculinity, women will identify negative experiences and outcomes [4]. Therefore, this study is supported by three fundamental theoretical contributions: the theory of tokenism [5], the conceptualization of organizations as gendered contexts [6], and the conceptualization of the “gender regime”, created by R. Connell [7,8].

### 1.1. Tokenism Theory: The Importance of Numerical Proportion

Rosabeth Moss Kanter [5] pioneered the study of group dynamics in organizations, having documented the inequalities existing between men and women. In her seminal study, she interviewed 20 women working in sales for a multinational company with a total of 300 employees, in addition to some of their colleagues and managers. Kanter [5,9] identified three factors which she perceived as disadvantages for these women: (i) the opportunity structure, whereby they gained fewer opportunities for training and career progression; (ii) the holding of organizational resources and power, whereby they gained less than men; (iii) and the numerical proportion, whereby they were outnumbered in the organization. This last factor became central to the development of Kanter’s [5,9] theory of tokenism.

The author identified four types of groups (uniform groups, skewed groups, titled groups, and balanced groups), focusing particularly on skewed groups, where, according to the author, there is an 85:15 ratio, with this dominance of one category over the other leading to discrimination. Kanter referred to the majority category as “dominant” in keeping with its control over the group and its culture, and the category making up the extreme minority as “tokens” as they are often treated as representative of the categories to which they belong and are therefore symbols or examples rather than individual persons. As the minority, tokens experience difficulties in developing bonds with other members of the extended group. Kanter [9] concluded that the “proportional rarity of tokens is associated with three perceptual tendencies: visibility, contrast, and assimilation” (p. 210).

Firstly, high visibility occurs due to the obvious or visible differences to others so that tokens, because they represent a much smaller numerical proportion in the group, attract more attention from other members, generating pressures for them to perform well. This may arouse the need to live up to expectations in tokens so they work harder than the dominant members to prove their abilities and be accepted as equally competent, but, contradictorily, such additional efforts may also lead to poor performance.

Secondly, according to Kanter [9], the contrast or polarization of differences between the tokens and the dominant group occurs on behalf of the latter, which can lead to the isolation or social exclusion of the tokens.

Thirdly, and finally, and to facilitate the process of acceptance in these contexts, there occurs the assimilation of the stereotypical roles of the group to which the tokens belong, and as attributed by the dominant group. “Role encapsulation” or “role entrapment” may thus occur, a kind of role imprisonment [9] (p. 212), with the tokens afraid to express any deviation to not contradict the expectations imposed by the dominant group and jeopardize their opportunities for integration into the organization. This inhibition of their authentic expression and anything that does not correspond to the stereotypical traits reinforces an identity constructed by the dominant group and, according to Kanter [9], may drive the further marginalization of the tokens.

As seen, Kanter’s theorization [5] begins with a study focused on women as a minority group before going on to generalize her results by positing that tokenism phenomena may occur in other situations with other under-represented social groups, which has since been subject to some criticism [10,11]. However, as far as women are concerned, there is certainly enough evidence to corroborate Kanter’s theorization [5,9] because, whenever constituting 15% or less of the total workplace population, women are considered tokens and tend to experience the associated consequences (e.g., see Santos and Amâncio 2014).

However, as Santos and Amâncio [3] emphasize in their study, Kanter [5] limited her analysis to the numerical proportion without, therefore, valuing the “structural, cultural, social and psychological factors, which may contribute to affect the interactions between groups and the performances of the symbols with the dominant ones in the organizations” (p. 708). Furthermore, Kanter’s [5,9] analysis focused on the organizational structure and did not conceptualize gender as an integral component of structure and hence fails to value her own observations on masculinity and organizations [6].

### 1.2. The Importance of Gender in Organizations

According to Acker [6], the structures of organizations are gendered; thus, they are constructed out of gender inequalities. Following their creation, these inequalities are then reproduced not only in the ways of organizing and exercising power but also in interpersonal relationships as the definition of work contains implicit preferences for male workers. In fact, for their positions of leadership and power, organizational structures seek people who ideally do not have family responsibilities and are correspondingly almost totally available to devote their time and attention to working. Combined with the emphasis on rational and strong leaders, this not only reflects gendered hierarchies but also simultaneously perpetuates their existence [12].

This gendering of organizations makes explicit that “advantage and disadvantage, exploitation and control, action and emotion, meaning and identity are patterned through and in terms of a distinction between male and female, masculine and feminine” [6] (p. 146). Thus, gender must be framed within processes, and attempting to undertake neutral analysis that fails to consider the other processes is inadequate [7]. Acker [6] identified five processes that reproduce gender divisions in organizations: (i) the division and organization of work; (ii) the construction of symbols and images of masculinity and femininity; (iii) the interactions between men and women, women and women, men and men, and all the patterns that represent dominance and submission; (iv) the production of gender components in each person’s individual identity; and (v) the interconnection of gender with the formation and development of social structure processes, including in the family and social spheres.

The work of Christine Williams [13] has highlighted how men and women are subtly socialized by a gendered professional ideology, leading her to claim that women tokens and men tokens face different consequences despite their shared tokenism and thereby contradicting the universality of Kanter’s perspective. In fact, in contexts of under-representation, while women encounter what is commonly called the “glass ceiling” [14,15], men are instead quickly “pushed” up the organizational hierarchy, a phenomenon Williams [13] labels the “glass escalator”. Thus, we should bear in mind that being in an extreme minority is not the only determinant of tokenism-related phenomena, hence the crucial need to consider other variables, such as social status [3].

Connell’s [7,8] conceptualization of the “gender regime” is, in this line of thinking, very relevant. As the “gender regime” encompasses the broad pattern of gender relations ongoing within an organization, which frame the context for specific individual events, relationships, and practices, this also extends to all the dimensions of gender relations distinguished by Connell [7]: (i) the gendered division of labor; (ii) gendered power relations; (iii) emotions and human relations; and (iv) gender culture and symbolism.

### 1.3. Football as a Universe of Men and Hegemonic Masculinity

The sporting context is broadly gendered even though different sports display different specificities [16,17]. Hence, the management structures and policies of sports organizations are not only male-dominated but also reproduce the traditional social attributions associated with their sex [18,19]. Furthermore, from a symbolic point of view, for example, the space occupied in the media, masculine sporting practices and their protagonists clearly predominate [20,21,22], which reinforces the strong link between sport and men but that does not get extended to women. This scenario has been the subject of debate with some countries taking measures to boost the opportunities for female inclusion and decreasing the factors still preventing this [23,24].

In the international context, the positions of manager, coach, referee, judge, and player, even with variations across the sports, have been and continue to be clearly and overwhelmingly occupied by men, and with the cultures based on this hegemony still prevailing [20,25,26,27].

Football is paradigmatic as a sport and context in which phenomena associated with male predominance and the symbolic domination of hegemonic masculinity emerge [25,28]. As a result of multiple factors [29], football represents a context for exercising masculine hegemony and correspondingly produces evidence both in terms of participation and support [30,31,32], as well as the performance of professional roles, such as board members, coaches, and club administrators [23,33].

According to Bromberger [34], the football universe mirrors very well how men are socially constructed, and, from school age onwards, the values of strength, competence, dexterity, and group solidarity are inculcated and internalized in boys. For boys and men, particularly adolescents in keeping with how they are more aware of the social expectations required to establish an authentic self, one notes a resonance in the narrative that violence and rigidity in sport are indicative of the natural predispositions of “real” men [35].

Playing football validates the vocal expression of the prerogatives of masculine “culture”, such as the right to express aggressive gestures, to deploy offensive terms and vernacular, and to jeer. Such behaviors, related to aggression, strength, and dominance, are perceived as predictors of success in football [36] and are almost exclusively linked to masculinity, so these “aggressive competitive dispositions are all too often rendered as natural traits” [37] (p. 167).

Tyler et al. [38] expanded the concept of “extremely gendered” organizations, hypothesizing that there are many more highly masculinized organizations beyond those in the military context, advocated as the only example by Sasson-Levy [39]. Thus, football emerges as an important context for the maintenance and reproduction of hegemonic forms of masculinity [17], representing one of the routes for the social construction of masculinity due to its combination of strength, competence, and power [40] and enabling the sport to play a crucial role in the protection and production of masculine predominance and in resistance to social changes towards equality [38].

The popularity of football as a mass activity partially derives from its glorification of masculine superiority so when women are not allowed to participate, they are relegated to an altered and assumed inferior form of the sport [20,28,41]. Hence, preserving the masculinization of football, through the removal of women from this sport, underpins the success of men’s leadership and primacy [25].

### 1.4. The Portuguese Scenario

As in many countries [20,25], in the Portuguese sporting context, there are also inequalities between men and women with regard to competition prizes, sports practice indexes, and the number of federated athletes (see Table 1).

The study by Cristina Almeida [43] demonstrated how women at that time were grossly under-represented in the sporting context, especially in decision-making positions, such as managers (around 11.5% women), with the percentage of women decreasing the higher the hierarchical position is. In the following decade, as the values in Table 2 show, the percentage of female directors in sports evolved positively but very slowly.

In terms of the functions of coach, referee, or judge, women have constituted and still constitute an extreme minority [26,27,33], a phenomenon very evident in the Portuguese reality as Table 3 mirrors.

The data presented for Portugal clearly demonstrate that the world of sport, despite slight and slow progress towards integrating women into various areas, remains a field in which males still utterly predominate. We assume that the specificities described in the previous item about the context of football (staff/non-players) may be similar in Portugal, which is why we chose it as the focus of interest in this study.

With this study, we intend to analyze and understand whether, in the specific context of football where women constitute a small minority (i.e., tokens), they experience the negative consequences identified by Kanter [5,9]. We take this theorization as our starting point but add on a gender perspective, specifically seeking to (i) identify the possible emergence of phenomena associated with tokenism [5,9]—the greater visibility, contrast, or polarization of differences and the assimilation of stereotypical roles—in integrating women in the football context; (ii) identify the eventual obstacles and difficulties experienced by women involved in the football context; and (iii) identify the strategies adopted by women involved in the football context to integrate themselves into a largely masculine-dominated context.

From a theoretical point of view, this study aims to contribute to the deepening of Kanter’s [9] theorization on the effects of tokenism in a specific context but recognizing the effects of gender. This will be possible and enriching through the conceptualization of organizations as contexts of construction and as evidence of gender inequalities [6] and the conception of the football context as a gender regime in which men and masculinity are, symbolically and effectively, dominant [7,17]. From the point of view of social change, the results of this study will potentially generate a reflection on the experiences of gender relations in the context of football, in Portugal, but also in culturally similar countries.

## 2. Materials and Methods

In this study, we adopted a qualitative approach, using a brief sociodemographic questionnaire and a semi-structured interview guide, because we think it allows us to better understand people’s beliefs, experiences, attitudes, interactions, and behaviors and thus better respond to the initial objectives.

### 2.1. Participants

In accordance with the data saturation and data sufficiency principles [45], we carried out 16 individual semi-structured interviews with 8 men and 8 women, from Portugal, aged between 21 and 59 years (M = 32.81; SD = 11.44). Among these 16 participants, two men and one woman were married, and the rest were single. More than half of the interviewees were university graduates (four men and six women), three held master’s degrees (two men and one woman), two males had secondary school education, and one held a post-graduate degree.

The essential inclusion criterion for participating in this research consisted of the requirement of performing functions in a football-related organization, whether in the fields of coaching, psychology, the medical team, planning, refereeing, or management. Of the sixteen interviewees, five (three men and two women) held managerial positions. Only two participants (one man and one woman) reported to female managers, with the remaining participants overseen by men. Over half (three men and six women) had been working in the football context for less than 10 years, with only one (man) for over 20 years. Due to the objectives defined for this study and its exploratory nature, in the selection of participants, we valued, above all, the advantages of diversifying their professional functions and, of course, the balanced representation of both sexes. Therefore, the length of their careers, as well as the level of prestige of the clubs in which they work, will not be subject to discriminatory analyses based on these differences.

### 2.2. Procedures

The process of recruiting participants was based on convenience as we initially drew up a list of positions of professionals working in football relevant for inclusion in this study, such as coaches, referees, technical coordinators, sports managers, and physiotherapists. Subsequently, we contacted persons known to the third author of this manuscript to inquire about their availability to participate in this study and sent informed consent forms to all potential participants. The response rate from those approached was 100%. This study met all the ethical principles of research and personal data processing (e.g., informed consent and debriefing, including information on the voluntary nature of participation, anonymity, processing and storing personal data, and confidential treatment of information). The informed consent was designed in accordance with the guidelines of the University Ethics Committee of the first author. In this way, we ensured that participants had full knowledge about the research objectives, were aware of the guarantee of confidentiality and anonymity, and gave their prior and explicit permission for the recording and subsequent transcription of the interviews.

All participants completed a brief questionnaire to collect sociodemographic data. The interviews were conducted between 17 December 2021 and 15 April 2022, always at agreed times in accordance with interviewee availability. Due to the consequences of the COVID-19 pandemic, and by the expressed wishes of the participants, who were probably still worried about the coronavirus, most interviews took place online, via the Zoom platform, with only four held face-to-face. The interviews took an average of 33 min. All interviews were conducted by the third author, as part of an academic study for a master’s degree in psychology. Therefore, the first author organized a training session for conducting interviews, followed by feedback, especially due to the need to opt for online interviews.

### 2.3. Instruments

We collected data through two instruments: a brief self-completion questionnaire and an interview guide. We chose to carry out interviews because this data collection technique has a high potential to respond to the objectives of this study, in the experiential and subjective domains, which we could only access through the narratives of women and men directly involved in the context. Indeed, the potentialities of the interview, namely with a semi-structured nature, are recognized when it is “intended to elicit views and opinions from participants” [45] (p. 188). The questionnaire was designed to collect sociodemographic data characterizing the participants and considered the following questions: sex, age, nationality, civil status, academic qualifications, year in which he/she started working in this profession, current place of work, function performed, department, and to whom he/she reports: Is it a man or a woman? For the semi-structured interviews, a guide with a version for women and another for men was built and followed, based on the previous literature review, and guided by the study objectives. Both guides contained questions, organized into four large dimensions: (i) factors that determine integration into football; (ii) gender inequalities in the context of football; (iii) the experiences lived by women in their current professional contexts; and (iv) strategies for managing the minority status of women. The formulation of the questions in the two guide versions differed, considering the focus and objectives of this study. In the context of football, women constitute the dominated group and men the dominant group, so women were asked about their experiences and men about their observations about women’s experiences.

### 2.4. Data Analysis

The text corpus resulting from the full transcription of the interviews was subject to thematic analysis [46], a methodological option justified by its suitability to the objectives formulated for this research and for allowing the identification, analysis, and summary of patterns/themes in discursive materials and, cumulatively, the achievement of in-depth descriptions, highlighting similarities and differences. Thematic analysis covers six distinct phases: (i) familiarization with the data; (ii) generating initial codes; (iii) searching for themes; (iv) reviewing themes; (v) defining and naming themes; and (vi) producing the final report; see [46] (p. 87).

In carrying out the analysis, we followed the steps proposed by the authors and carried out what they call ‘mixed’ analysis [46], namely, inductive and deductive. Thus, we started our analysis (the phase of searching for themes and sub-themes) according to a deductive orientation, considering the three essential study topics—visibility, polarization, and assimilation [5,9]—and, therefore, of the interviews. Nevertheless, we simultaneously allowed for the eventual identification of unexpected but pertinent themes and/or sub-themes, having thus assumed an inductive analytical positioning. This analytical strategy was carried out among the research team to ensure that the themes provided a rich description of the whole body of data and to increase the guarantee of the validity and reliability of the analysis. The results are presented in the following section.

## 3. Findings

The analysis allowed us to identify the six themes presented in Table 4, designated as follows: (i) integration into football: influences, support, and obstacles; (ii) football as a masculine context: determinants and arguments; (iii) the visibility of women in football; (iv) the polarization of differences between women and men in football; (v) assimilation to stereotypical roles of women; and (vi) women’s strategies in managing their token positions.

### 3.1. Integration into Football: Influences, Support, and Obstacles

This theme approaches the means of integration into football and is subdivided into three sub-themes: (i) family influence; (ii) social support and assistance; and (iii) the obstacles and challenges encountered.

In the first sub-theme, the integration of interviewees into the world of football emerges as largely due to family influences, specifically from fathers who already played the sport, and ensured the participation of their children in sports organizations, as illustrated by the following excerpts:

*My father is a football coach, although I never played football. I come from a handball background, but football came into my life many, many years ago. (I2, woman, 29 years old, Psychology, 6 years of experience)*

*I played, then my son started to play, and I accompanied him to games and training. And then a coach invited me; there are no people for this function. (I8, man, 51 years old, Planning, 16 years of experience)*

With regard to the sub-theme of social support, interviewee opinions diverge by gender. Women highlight the lack of support from family and friends, their fears regarding their professional future, and an awareness that football is a male-dominated context. Men, on the other hand, report greater encouragement and support as their process of integration into the sporting context was considered natural and even expected by family and friends:

*At the time, they called me crazy, a woman sitting on the Board was unthinkable, that we didn’t understand football, we didn’t understand anything (....). But then people get used to it. But it was the men, which is a more complicated issue, be-cause the parents... Women on a Board, ahh, but what does she really know about football? What has she come here to do? (I4, woman, 51 years old, Planning, 11 years of experience)*

*[Reactions] They were good. In my family, football was no stranger. In relation to my group of friends at the time, it wasn’t either because we all liked football. So, it was something natural and very well accepted. (I3, man, 32 years old, Coaching, 15 years of experience)*

Regarding the subtheme on the obstacles or challenges encountered when entering the footballing context, once again there is a dichotomy in the perspectives of women and men. The women referred to the difficulties caused by the fact that football is a male-dominated world, highlighting the prejudices and stereotypes associated with this universe. Most male respondents did not report experiencing adversities as many of them had previously been players, which facilitated their process of integration, albeit into other functions:

*(...) [I felt difficulties] because I was a woman, because it’s that taboo, and the world is still a bit macho, that women don’t belong in football, basically, and that the kids only went to the medical post because we women were there. (I15, woman, 29 years old, Medical Team, 7 years of experience)*

*(...) I had already been a football player, so I was in my own environment, right? So, that wasn’t new to me. It was new in the sense that I had the responsibility of leading a process, just that process, in which the players would already see me not as a teammate but as part of the group leadership. I didn’t feel any restrictions, I was also very well received by the structure, by the head coach at the time. (I12, man, 39 years old, Board member, 19 years of experience)*

### 3.2. Football as a Masculine Context: Determinants and Arguments

The second theme reveals the awareness among interviewees that football represents a markedly masculine context, highlighting the various factors that contribute to its persistence, as revealed by the following sub-themes: (i) progress and resistance; (ii) conflict between professional and family lives; (iii) women’s interests and opportunities; and (iv) difficulties with infrastructures.

The first sub-theme aggregates ideas that emphasize the greater gender balance in the footballing context, with reference to the increasing number of women, both playing the sport practice and in organizational infrastructures. However, there is a consensus that this progress is still insufficient as, despite efforts to include girls and women, the belief that football is for boys and men still prevails. Furthermore, the predominance of men and the low presence of women are also explained by reference to a lack of “training levels” in women’s football, hindering the continuation of learning for girls who want to become players and preventing the strengthening of the quality of women’s football:

*We have progressed very little. I mean, 10 years ago, to see women in football was something rare and unthinkable, now we are starting to see it: But, even so, it is incomparably less than the number of men, and I think this starts at the youngest ages. In other words, a boy who is five or six years old and wants to try a sport goes to football and a girl who is five or six years old and wants to try a sport goes to skating or dancing. (I3, man, 32 years old, Coaching, 15 years of experience)*

*That is, right now, there aren’t as many female coaches, quality players, as much as there are men because men have the foundations. That is, women are seen to have to start their work in senior football. And there, it becomes much more difficult. Right now, there is still an imbalance in the level of competences (...), if there is no development of women’s football training, there cannot be equality. (I1, man, 23 years old, Coaching, 8 years of experience)*

*(...) at this moment, it is a world that is still growing and still with a lot to grow. There are still many obstacles that I try to knock down, as a woman (...). And it was still a bit like that, it still is, it is still like that, women do not belong to football. But now, throughout this year, it’s growing even more. (I15, woman, 29 years old, Medical Team, 7 years of experience)*

A second identified sub-theme reveals another reason justifying the reduced number of women in football: the difficulties in reconciling professional and family lives. The participants assume that women continue to perform the bulk of the domestic tasks and provide family care and their dedication to football would hence be compromised, both as spectators and participants. Therefore, the issue of availability is pointed out as a conditioning factor for the inclusion of women in football:

*I think that men still dominate a lot of things and I think we are a very macho society. (...) And today, it is not quite like that, because a situation also prevails in many homes, some families, is that the man tends not to take care of the children, when there is any illness, it is always the woman who is absent from work. (I4, woman, 51 years old, Planning, 11 years of experience)*

*A woman has a lot more to do at home’ I’m not macho, but at home, what my wife does versus what I do, I’m much freer for other functions. And I think that, then, when a woman decides to start a family, she abandons sports completely. (I8, man, 51 years old, Planning, 16 years of experience)*

Another sub-theme identified focuses on other factors that, according to the participants, help explain the predominance of men and the rarity of women in football. These interviewees point to women’s lack of interest in sport and football as a justification for their under-representation, especially in management and positions of responsibility. This argumentative version, based on women’s accountability, coexists, however, with one that focuses on ideologies and practices in the footballing context. In effect, the low presence of women in this context is, according to some participants, due to the lack of opportunities for them to access positions in the football context as an effect of the persistent belief that football is only for men, which underpins the maintenance of effective barriers to their participation and involvement:

*Well, we have more men than women working here, that is a fact, but I don’t see this as something that has been done on purpose. I think that women have less interest in this part of football; even at the board level, at the managerial level, I still see few women in this world. (...) I think that opportunities exist, (...) and that the doors are open. We do not close doors to recruiting women, and I am talking very specifically about our context. I do not know what is happening within other clubs, but I think that this is really because of the lack of interest of women. (I12, man, 39 years old, Board member, 19 years of experience)*

*I think that it is also due to stigma. It is not that there are no good women players with a future and potential but maybe there are not so many opportunities; therefore, men have some advantages in these offers (...) It is not exactly a question of there not being women who like sport or football, but when they do, there are fewer opportunities for them to follow this path and not so much incentive from people, municipalities and clubs (...). (I7, woman, 23 years old, Psychology, 1 year of experience)*

The last sub-theme organizes the ideas expressed about the difficulties posed to the presence of women in football contexts and which result from specific infrastructures and logistical and organizational factors. According to the interviewees, the frequent absence of support facilities (changing rooms, toilets, etcetera) for women in many sports organizations represents not only a constraint but may also even lead to their exclusion. Apart from having to wait for male colleagues to leave in order to enter, female coaches do not have opportunities to participate in the conversations and dynamics taking place among their male colleagues inside locker rooms:

*It is, for example, clear that they cannot change into equipment in the same spaces. Those conversations that you have in the locker room, outside the training, exist and create some disadvantage for the woman because the relationship will never be as good. Women are not there in the most intimate part, in the most intimate conversations that, at this moment, are still inside the locker room, or leaving the locker room. There is still a difference. (I1, man, 23 years old, Coaching, 8 years of experience)*

*What I still find, nowadays, is, for example, as I work mostly with men, when we go outside the facilities where I work, and this comes from way back, we don’t exactly have facilities for us. You go to an away game, a ladies’ locker room doesn’t exist, you want to go to the bathroom, you must wait until everyone else has left so you can enter. (...) I have a lot of difficulty if we go to an away coaching course, (...) because it involves a room for only one woman, or you don’t go because you can’t enter the locker room or there is a crisis, and you can’t enter the locker room because everyone is undressed. (I2, woman, 29 years old, Psychology, 6 years of experience)*

### 3.3. Visibility of Women in Football

The third theme identified spans the phenomenon around the greater visibility of women in football that breaks down into two sub-themes: (i) the greater exposure of women and (ii) the pressure on women for evidence of competences and contextual mistrust.

According to the interviewees, as the number of women in football is so small, they are always left more exposed and stand out, for example, whether in competitions or coaching sessions. Participants also refer to how it is easier to meet every woman and know their names in football organizations, precisely because of their reduced number:

*We are few and, when we are so few, we must... it’s not learning, but being aware to this issue of “we’re going to make an impact and people are going to know who we are”. (...) A woman, everyone knows that that woman is V or J, right? (I2, woman, 29 years old, Psychology, 6 years of experience)*

*(...) the most exposed end up being the women, because they are a smaller number of members, regarding football, they always end up being considered the weakest link. (I10, man, 25 years old, Coaching, 8 years of experience)*

The second sub-theme organizes the interviewee perspectives on the surveillance of the performance of women in football. The participants state that women, more than men, are called upon to prove their competences and must correspondingly apply more effort to perform well and prove they are worthy of the opportunities granted. One of the reasons pointed out for this phenomenon, felt by women and perceived by men, is the initial mistrust and doubts about women’s effective competences to get involved in this environment, which are founded on the belief that only men naturally understand football and are prepared to get really involved in it:

*I think so [they feel more need to prove their competences and feel pressure to perform better], because the leader is a man. They may even want to be leaders one day, but they are not leaders because that opportunity has not yet appeared. And so, I think that they will always be fighting for that opportunity and, maybe yes, I’m not in their heads, but they will want to do everything perfect, be more committed and want to show more service, because they think they may even be prepared to have other functions and want to be prepared for when that opportunity arises. (I1, man, 23 years old, Coaching, 8 years of experience)*

*Sometimes, they start to drop hints before the match starts (...). So (...), we try to be more focused, more attentive to the shots, so that we don’t make mistakes and prove them wrong, that we even exceeded their expectations of us, both the public and the teams. (I13, woman, 25 years old, Refereeing, 7 years of experience)*

*(...) I always try to do a good job and demonstrate my ability, that is, I always take an injury that supposedly takes x amount of time and I try to shorten it so as to impress and, at the same time, to test myself, I don’t know if it makes sense, but I always end up doing this. (I15, woman, 29 years old, Medical Team, 7 years of experience)*

### 3.4. Polarization of Differences between Women and Men in Football

The fourth identified theme frames the polarization of differences between women and men by the latter and is structured into three sub-themes: (i) differentiated treatment of women; (ii) negative discrimination against women; and (iii) sexual harassment.

Generally, both participant genders agree that women and men are treated differently to the detriment of the former in football and are adamant about practices of negative discrimination existing towards women:

*I never felt it. But, yes, I am aware that many women are treated differently, especially if they occupy coaching roles. Often the parents in the stands say: ‘she’s a woman, she doesn’t understand any of this’. Yes, there is still a lot of that kind of discrimination and prejudice. (I14, woman, 34 years old, Board and Medical Team, 11 years of experience)*

*[Women in women’s football] are still treated differently. We can speak about the salary issue; we can speak about the issue of the timetables for their training. Why do men train earlier, and women train later? (I1, man, 23 years old, Coaching, 8 years of experience)*

Participants of both sexes illustrate their perceptions about the negative discrimination against women in football. However, it is women who make most recourse to personal experiences regarding their difficulties whether in occupying management or leadership positions in sports organizations or in participating in informal moments of conviviality, in addition to direct verbal criticism, sometimes violent, for their presence in a traditionally male-dominated context:

*As for female referees, I’ve already felt [discrimination against them], but you tell me like “ahh, but male referees also get insulted a lot and called many names” and it’s true. Here, (...) it’s instead of calling me names, instead of the public insulting, the public will simply say (...) “ahh, because this is not for women, football is for men”. (...) It’s practically the same thing, in little heads it is “football is not for them”. (I3, man, 32 years old, Coaching, 15 years of experience)*

*I was on the way out of my old club. (...) I was, okay, inclined towards whether I was going to leave. But as it was a club I’d been at for many years, I heard the proposal from the new president (...) and I am ambitious. In the world of football, I was expecting to go up, right? And the proposal was completely the opposite and I asked why, and he said it was because a new coach was coming who didn’t want women. And I asked how come he didn’t want women? (...) So, I was totally discriminated against. I couldn’t move up as a new person was coming in, with other better functions, whoever he is, for a position higher than mine because he is a man, and I am a woman. (I15, woman, 29 years old, Medical Team, 7 years of experience)*

*Always hearing those comments that “you might as well be at home making lunch or dinner”, those stereotypes they create about women in their day-to-day lives. In the game, no, it’s that discrimination, it’s always those comments they make, like they tell us, they refer to us as being inferior (...). (I13, woman, 25 years old, Refereeing, 7 years of experience)*

The third sub-theme aggregates the references to episodes of sexual harassment described by the women interviewed, the protagonists of which were colleagues or players, and which gave rise to experiences that were difficult to cope with:

*I know that when there are male coaches who are macho towards women and treat them badly, and they don’t like it and then they have that bad atmosphere and leave or because they are harassed and then they leave. This happens a lot in women’s football. And there is not only harassment from men, there is also harassment from women, that exists in both contexts. (I4, woman, 51 years, Planning, 11 years of experience)*

*I felt that, for example, I had to pass through a corridor to gain access to the fields where I followed the two teams I was supervising and when I went along there, I had the feeling everyone was looking, someone would make some kind of leering comment, they would laugh, for example (...). I felt some .... I felt .... harassment, let’s say, yes, harassment and some attempts to overstep some barriers, to separate what was the work that I was doing there as a psychologist, trying to push me more in personal terms. (I7, woman, 23 years old, Psychology, 1 year of experience)*

*But harassment, yes, in the games, the players of opposing teams too, and always the opposing team’s squad, during match times. (I15, woman, 29 years old, Medical Team, 7 years of experience)*

### 3.5. Assimilation to Stereotypical Roles of Women

The fifth identified theme deals with the phenomenon around the assimilation of stereotypical roles by women, which encompasses three sub-themes: (i) male paternalism; (ii) femininity and maternal roles; and (iii) the organization and rationality of women.

The first sub-theme organizes the ideas of interviewees of both sexes on how women are treated in this context and the underlying justifications. These ideas convey the expression of paternalistic attitudes by men towards women on the grounds of feeling the need to protect them, to adopt and display more careful language on the assumption that women are more fragile and sensitive, and the lack of all the competences for performing their functions in the football context:

*(...) when there’s a more aggressive subject, or some feedback is more skewed, or something explodes, everything is always very “ok, sorry, I didn’t want to tell you this; calm down, guys, there’s a woman there, be careful”. So, they are always careful, they don’t come at us. For example, they never came at me, exactly, screaming, (...) they have another way of talking to us (...). They know that women do not deal very well with screaming (...), we, particularly, associate ourselves with this stigma of emotionality and fragility (...). (I2, woman, 29 years old, Psychology, 6 years of experience)*

*I was a little more careful (...), because we are always afraid of some comments from some kids, we are always afraid, and we tend to protect them a little bit (...). Well, we must be careful, it’s a woman, she’s not prepared, she’s more fragile, we can’t scream, or we can’t make more aggressive approaches. (I3, man, 32 years old, Coaching, 15 years of experience)*

Aligned with the first sub-theme, the next one aggregates interviewee ideas about some traits of women in football that seem to describe a maternal figure, such as affective expressiveness, protection, and the provision of emotional comfort:

*Women are more sensitive, more observant in terms of human relations, men neglect this. They are people who are more open, more concerned. It is much easier for our athletes to feel more comfortable when they are talking to a woman than a man because they always see man as the authority, so they are always, or at least most of the time, embarrassed. (I12, man, 39 years old, Board member, 19 years of experience)*

*At the main training levels, they have a different connection with us than with men, because they look at us as more maternal figures and we end up fostering more complicity with them. In situations of greater stress, they manage to calm down more with us women than they do with men (...) (I14, woman, 34 years old, Board and Medical Team, 11 years of experience)*

Finally, the last sub-theme interlinks the opinions describing women in the footballing context as possessing organizational, pondering, self-control, and appropriate emotional management competences in contrast to men, who are less operational and more emotional:

*[Men] are very practical and sometimes they get carried away by their emotions. They don’t think about things very much and we women have the capacity of “ok, calm down, let’s get down, sit down here, let’s talk about it and work”. We are much more operational and have a working capacity that men often don’t have. They are more disorganized and get lost (...) (I2, woman, 29 years old, Psychology, 6 years of experience)*

*We are much more organized. We are also, on the one hand, emotional, but on the other hand, we are much more rational. We know that there is a problem. Ok, let’s look at the problem, let’s think about what is needed or what is not and what we must do. And they, sometimes, especially in the football business, it’s very much the heart. In fact, men are much more carried away by emotions than women in that sense. (I11, woman, 21 years old, Coaching, 1 year of experience)*

*There is the issue that women enter a context in which they are normally more intelligent than men, because they expose themselves less to criticism, because they are more observant. Men tend, at certain moments, to talk too much and then it is the ego. I don’t know, maybe it is the male hormones that speak louder. Women are more rational, in that sense. (I12, man, 39 years old, Board member, 19 years of experience)*

### 3.6. Women’s Strategies in Managing their Token Positions

The sixth and last theme resulting from our analysis focuses on the strategies adopted by women to manage their presence in contexts where they are a numerical minority. This theme contains two sub-themes: (i) commitment and adaptation; and (ii) demonstration of professionalism.

The first sub-theme brings together ideas that describe women’s adaptive strategies to ensure their integration into the environment through their selection of language and behavior and by the demonstration of competences:

*(...) Some strategies of being careful about what I’m going to wear. (...) Part of my common sense, which is if I’m one of the few women who is in the middle of men, I don’t go in short shorts just out of respect for myself and my body, because I know it’s going to be a reason for conversation. (I2, woman, 29 years old, Psychology, 6 years of experience)*

*But I think we must take a stance, change the stance dependent on the places and it’s not because you are in football, you change to the stance of “ok, this is sport, I have to be a bit harder”, but smile and do everything as if you were a woman. (I4, woman, 51 years old, Planning, 11 years of experience)*

*(...) the woman herself worries about what the other person thinks of her and leads to her actions sometimes being studied, (...) for fear that it will lead to other interpretations. (I8, man, 51 years old, Planning, 16 years of experience)*

In addition to commitment and the adoption of strategies to adapt to the masculine context, the need to demonstrate professionalism was strongly referenced by interviewees. This constitutes the second sub-theme and alludes to the separation between personal and professional lives, the appropriateness of their presentation, and their dedication to work:

*I don’t go to dinner every day with my work colleagues. I am professional and I am a person and I do not mix work with the personal dimension. (I2, woman, 29 years old, Psychology, 6 years of experience)*

*I think the strategies they adopt is to work, that is, maybe they think that more work is needed to be recognized, not to fail at any moment, to try to balance things and be more seen, more valued (...). (I16, man, 30 years old, Refereeing, 3 years of experience)*

## 4. Discussion

This study of women’s experiences within Portuguese football organizations was guided by the theorization of Rosabeth M. Kanter [5,9], given that they constitute members of token groups. Indeed, in Portugal, as in most countries, women involved in sporting contexts, and particularly football, represent a minority and occupy functions socially dominated by men and bound up with hegemonic masculinity [20,30,32].

We delimit football, and the functions of manager, coach, referee or line judge, and medical team members in this sport, as the context for observing any evidence of the tokenism-associated phenomena as identified by Kanter [5,9]. Thus, as a central objective, we sought to identify the eventual emergence of negative consequences from the token position held by women, specifically high visibility as a member of a dominated group, polarization in their differences, and their assimilation of stereotypical feminine roles. Cumulatively, we tried to identify the difficulties and strategies of the inclusion and adaptation of women in the football context while also attributing meanings to the perspectives of men in this context as members of the dominant group.

One of the negative consequences for people in token situations arises from their greater visibility [5,9]. Based on our analysis, there is no doubt that women engaging with the football universe are manifestly more visible, which is confirmed by participants of both sexes. In the understanding of these interviewees, this fact stems from the scarcity of the number of women which, in turn, derives from their shallow interest in sport and the difficulties in conciliating these activities with family life. The greater visibility almost directly associated with minority groups of women has been widely demonstrated in various professional and organizational contexts, e.g., [2,38,47,48], and this study represents a contribution to these findings.

The greater visibility of the tokens and their consequent need to constantly demonstrate their competences and counteract low expectations of good performance standards [5,9] are, we believe, predictable for women in the footballing universe in keeping with its specificity. Indeed, this study identifies how the greater visibility of women generates constant surveillance of their behaviors and specific competences as they are involved in an environment which has always been dominated by men and which, according to prevailing social perceptions, naturally belongs to them. This confirms both the findings of studies in the same context [49,50] and other professional contexts, e.g., [47,51].

The polarization of the differences between the members of the dominant and dominated groups, through actions taken by the former, is, according to Kanter [5,9], another of the negative consequences borne by the tokens. Our analysis allows us to confirm that this phenomenon emerges in the context in question: men and women, in exercising their functions, are treated differently, with a clear emphasis on what is appropriate to each sex based on arguments around the social gender constructions.

Interviewees of both sexes recognize how women are subject to negative discrimination in this context, expressed, for example, in the difficulties in accessing management positions, career progression, and lower salaries as identified, especially regarding female coaches [50] but also in management positions in this sport [25]. In our analysis, we consider that this represents a disadvantageous situation for women and hence the application of the glass ceiling metaphor [14,15]. Our analysis also allows us to defend the application of the “concrete ceiling” metaphor, as used by Leanne Norman and colleagues [52], as this appropriately depicts the resistance of male predominance and hegemony to the full integration of women into several football functions.

Through the words of participants of both sexes, we highlighted how very common it is for women to be excluded from informal sports team events; that they are victims of verbal abuse, particularly from the public, the families of players, and opposing teams; and that they also feel the need to ensure that their own appearance and that of their players is “discreet”. Furthermore, and more disturbingly, this study also indicates the occurrence of sexual harassment of women involved in the context of football, alluded to and narrated with some subtlety and withdrawal on their part.

Our observations corroborate the contributions of other studies, especially regarding the marginalization of women, through their circumscription to “peripheral” domains and spaces to maintain the gender hierarchy and the culture of male hegemony [25], with the eroticization of their bodies also serving as a means of diminishing their agency and confirming their unsuitability for the sports universe [53,54].

The women participating in this study underline the difficulties of maintaining closeness with male players: “women are not there in the most intimate part, in the most intimate conversations”, which take place in the locker room. The common images of communication about conviviality (including victory celebrations) are, we think, very representative of how these spaces and contexts are occupied only by men. Generally, we think about, in this specific domain, one of the conclusions of the study by Bryan et al. [25] (p. 962): “proximity to the male athletes and the field of play underpins a gendered substructure that distances women from core leadership roles by limiting their inclusion to roles that are peripheral to the organizational core”. In addition, in our analysis, and valuing the easily observable empirical elements, the images of men (players, managers, coaches, technicians) are the most evident and disseminated, which can be interpreted as a path for the construction of symbols and images of masculinity and femininity, according to Acker [6], as one of the processes that reproduce gender divisions in organizations.

The assimilation of stereotypical roles is, for Kanter [5,9], the last negative consequence of the token situation and corresponds to one of the themes of our analysis. The narratives of both sexes coincide in the evidence that men tend to be protective of women in the football context, resorting to more careful language and respectful attitudes towards the “sensitivity” and “fragility” they deem intrinsic to females. Such special attentions also receive justification by male respondents on the grounds of considering the lesser competences of women to exercise football functions. We consider that these narrated behaviors do not incorporate egalitarian motivations, as they reflect paternalistic motivations related to benevolent sexism [55].

Male participants express the conviction that women in this environment innately possess advantageous relational competences through being less authoritarian and more willing to listen, above all, in their relationships with athletes. The women, in their turn, confirm the existence of these expectations about their behavior and, in their own words, recognize themselves in them. This thereby also confirms how the desirability of and adaptation to traits of an idealized femininity, which emerges in other social contexts [3,47,56], are also present in this context.

The descriptions participants adopted to describe the men and women involved in football insistently refer to women’s abilities to organize work and manage emotional impulses and conflicts while also characterizing them as “more rational”. Correspondingly, there are also references to how men are more “disorganized” and tend to react “with their hearts” and also under the effect of “male hormones”. Such descriptions may mean that some specific contextual reasons may generate a departure from the stereotypes associated with each sex as identified in relation to female coaches [57], who may reproduce traits of stereotypical masculinity.

Our analysis, however, confirms the presence of the assimilation of stereotypical roles by women and the respective attribution by men in this respective context. Indeed, the capacities of women to organize and manage emotions and conflicts seem to make up for the absence (or lower levels) of these capacities in men. In our view, this places women in the position of careers, those who take care of tasks and functions that men may not perform, without this necessarily being detrimental to the assessment of their professional competences within this scope. We therefore consider that women in the football context are subject to “role entrapment” [9], as there is an overlap between what is socially and generically attributed to them as the ideal woman and the ways of being they should fulfil in the specific context of football.

In the same sense, in our opinion, the aforementioned levels of greater male emotionality should not be confused with the traits of femininity socially attributed to women [58,59]. In fact, this is not about the demonstration of emotions in positive, empathic, and altruistic relational moments—as we have seen—expected and desirable in women within this environment. Rather, these emotions displayed by men seem to be associated with expressions of the dominant masculinity, in this case, through aggressiveness and the demonstration of “natural” power, a means of affirming the prerogatives they benefit from in the football context [36,37,53].

The proportion of women in the various areas and/or functions related to Portuguese football, in the areas of coaching, psychology, the medical team, planning, refereeing, and management, has remained relatively stable: a small minority. Even so, many women join, integrate, and remain in this universe represented and experienced as a large “extremely gendered” organization, contrary to the idea of Sasson-Levy [39] that only military contexts would be suitable for this concept. Indeed, as Amée Bryan and colleagues [25] set out, in the football context, the exclusion of women is not formalized, and their segregation will be lesser than in military contexts, but both contexts display “a high degree of legitimacy for gender inequality” (p. 945).

## 5. Conclusions

Our study provides insights into the admission, permission, and scope of inclusion of women in the world of football as well as into the incessant insistence on demarcating differences and asymmetries between the most valid and desirable competences and traits (those of men). This interactive process ensures the maintenance of inequality affecting women [6] and, in this case, as a permanent and distinctive feature of football’s organizational culture.

In this environment, men do not seem ostentatious either in their delimiting of spaces of domination or in expressing displeasure over the presence of women. Women, in turn, seem to accommodate to the prevailing male norms, one of the viable strategies for managing daily life in a universe in which numerical superiority and the effective and symbolic domination of men are extremely present and assumed [23]. The adherence to traits associated with femininity, such as the “organization of the disorganization of men”, the reduction in interpersonal tensions, and the emotional care of athletes, may also be interpreted as the delimitation of a specific space for women, a second strategy for inclusion which, according to Jo Welford [23], women make recourse to in the context of football.

We consider that our results, although referring to a circumscribed regional context, allow us to state that football is constituted as a gender regime, in the sense attributed by Connell [8]. Indeed, we clearly signal the sexual division of labor between men and women—the asymmetrical division of power between both—namely due to their hierarchical positions and gendered occupations, interpersonal interactions and emotions marked by rigid stereotypes, and, finally, cultural and symbolic representations supported by resistant beliefs and attitudes about gender differences.

In this scenario, the legitimization of inequalities that affect women is ensured by subtle and hegemonic processes, and these inequalities do not reach high levels of visibility. According to Joan Acker [60], in such conditions, it will be less likely that organizations in which these phenomena occur will undertake effective change processes towards gender equality and equity.

### Limitations and Future Directions

Due to its objectives and theoretical framework, this study is inaugural in Portugal and should be assumed as exploratory and, therefore, with easily identifiable limitations: First, because its results, although relevant and valid, derive from the oral expression of a very small number of participants. Secondly, in line with our objectives and with the epistemological and methodological options, we did not try to ensure that the participants represented the multiplicity of characteristics of the professionals involved in the context of football. Therefore, the lack of stratification of the sample (considering, for example, ages, years of experience, type of club, and geographic location) makes it impossible to extrapolate the results to the universe in question. Thirdly, even if this is not exactly a limitation, the characteristics of qualitative studies must be considered. Indeed, in these studies, an attempt is made to know and/or understand a certain reality (the experience, the ideas, the events) based on the expression of the participants, assuming their subjectivity. Therefore, such “limitations” are intrinsic and refer to the impediment of safe generalization and projection, as occurs in many studies of a quantitative nature.

Despite the recognized limitations, we consider that this study shows the need to deepen the theme of the inclusion of women in the universe of Portuguese football and, thus, contribute to greater levels of knowledge and questioning. One of the ways of deepening this study could be through an equally qualitative study, but in order to overcome the first two limitations mentioned. That is, the amplification, diversification, and stratification of the set of participants would give robustness and comparability to the collected data and, therefore, to the results and conclusions. To answer more specific questions and more ambitious objectives, we believe that the adoption of “methodological triangulation” could be another option for the continuity of this study. Indeed, the deliberate and theoretically relevant articulation of quantitative and qualitative data would undoubtedly reinforce the comparability, depth, and complexity of analyses and knowledge about the context in question.

However, despite the assumed limitations, this pioneering study in Portugal can spark debate on the topic, particularly in its presentation at scientific events and in specific training sessions. Promoting the debate about women non-football players can be associated with the public and institutional debate about the inequality of treatment and resources of women’s football teams, with a lot of visibility and awareness, namely, with the participation of the Portuguese team at the 2023 FIFA Women’s World Cup.

## Figures and Tables

**Table 1:** Women in federated sports.

Years	Total	Women (%)
2003	376,465	18.6
2007	484,090	22.4
2011	523,168	25.2
2015	566,366	27.5
2018	667,715	30.4
2020	587,812	27.9
2021	483,829	29.6

Source: PORDATA [42].

**Table 2:** Women directors in sports.

Years	Total	Women (%)
2013	16,509	8.1%
2014	16,058	8.5%
2015	28,948	8.8%
2017	16,378	8.4%
2018	6309	9.4%
2020	5979	9.6%
2021	3740	10.0%
2022	4377	11.1%

Source: IPDJ, I.P., Federated Sports Statistics [44].

**Table 3:** Women coaches and referees or judges.

Functions	Years	Total	Women (%)
Coaches	2013	5639	1.6%
	2014	5895	1.4%
	2015	6427	1.3%
	2017	6679	2.5%
	2018	7061	2.8%
	2020	8623	4.2%
	2021	6690	7.2%
	2022	9978	4.5%
Referees or judges	2013	3977	6.2%
	2014	3383	6.0%
	2015	4370	8.4%
	2017	4186	7.5%
	2018	4780	8.1%
	2020	3967	9.0%
	2021	3713	12.7%
	2022	4256	10.5%

Source: IPDJ, I.P., Federated Sports Statistics [44].

**Table 4:** Themes and sub-themes.

Themes	Sub-Themes
Integration into football: influences, support, and obstacles	Family influence
	Social support and assistance
	Obstacles and challenges
Football as a masculine context: determinants and arguments	Progress and resistance
	Conflict between professional and family lives
	Women’s interests and opportunities
	Difficulties with infrastructures
Visibility of women in football	Greater exposure of women
	Pressure on women for evidence of competences and contextual mistrust
Polarization of differences between women and men in football	Differentiated treatment of women
	Negative discrimination against women
	Sexual harassment
Assimilation to stereotypical roles of women	Male paternalism
	Femininity and maternal roles
	Organization and rationality of women
Women’s strategies in managing their token positions	Commitment and adaptation
	Demonstration of professionalism

## Data Availability

The data presented in this study are available on request from the corresponding author. The data are not publicly available due to ethical restrictions.
